# Impact of the Reverse 2-Step Algorithm for *Clostridioides difficile* Testing in the Microbiology Laboratory on Hospitalized Patients

**DOI:** 10.1093/ofid/ofae244

**Published:** 2024-04-27

**Authors:** Evann E Hilt, Byron P Vaughn, Alison L Galdys, Michael D Evans, Patricia Ferrieri

**Affiliations:** Infectious Diseases Diagnostic Laboratory, Department of Laboratory Medicine and Pathology, University of Minnesota Medical School, Minneapolis, Minnesota, USA; Division of Gastroenterology, Hepatology, and Nutrition, University of Minnesota Medical School, Minneapolis, Minnesota, USA; Division of Infectious Disease and International Medicine, University of Minnesota Medical School, Minneapolis, Minnesota, USA; Clinical and Translational Science Institute, University of Minnesota, Minneapolis, Minnesota, USA; Infectious Diseases Diagnostic Laboratory, Department of Laboratory Medicine and Pathology, University of Minnesota Medical School, Minneapolis, Minnesota, USA

**Keywords:** antibiotic stewardship, *Clostridioides difficile* infection, enzyme immunoassay, molecular diagnostics, nucleic acid amplification tests

## Abstract

**Background:**

Multistep laboratory testing is recommended for the diagnosis of *Clostridioides difficile* infection (CDI). The aim of this study was to present the impact of multistep CDI diagnostic testing in an academic hospital system and evaluate the toxin B gene polymerase chain reaction (PCR) cycle threshold (Ct) values of PCR-positive tests.

**Methods:**

In October 2022, our system began reflex testing all PCR-positive stool samples with the *C. DIFF* QUIK CHEK COMPLETE (Techlab), an enzyme immunoassay–based test with results for the glutamate dehydrogenase antigen (GDH) and *C difficile* toxin A/B. Hospital-onset (HO) CDI and CDI antibiotic use before and after testing were tracked. Ct values were obtained from the Infectious Diseases Diagnostic Laboratory. Receiver operating curve analysis was used to examine the sensitivity and specificity for identifying GDH^+^/toxin^+^ and GDH^−^/toxin^−^ at various Ct thresholds.

**Results:**

The HO-CDI rate decreased from 0.352 cases per 1000 patient-days to 0.115 cases per 1000 patient-days post–reflex testing (*P* < .005). Anti-CDI antibiotics use decreased, but the decrease was not commensurate with CDI rates following reflex testing. PCR^+^/GDH^+^/toxin^+^ samples had a lower mean Ct value than PCR^+^/GDH^–^/toxin^–^ samples (23.3 vs 33.5, *P* < .0001). A Ct value of 28.65 could distinguish between those 2 groups. Fifty-four percent of PCR^+^/GDH^+^/toxin^−^ samples had a Ct value below that cut-off, suggesting the possibility of CDI with a negative toxin test.

**Conclusions:**

Reflex testing for a laboratory diagnosis of CDI results in rapid, systemwide decreases in the rate of HO-CDI. Additional research is needed to distinguish CDI from *C difficile* colonization in patients with discordant testing.

## Teaser

Implementing reflex testing for *Clostridioides difficile* infection (CDI) diagnosis significantly decreases hospital-onset CDI rates, but antibiotic use reduction was only partially attenuated. Discordant CDI testing patterns are common and additional strategies are needed to make an accurate CDI diagnosis.

*Clostridioides difficile* accounts for the highest proportion (15%) of hospital-associated infections in the United States [1, 2]. With an estimated 223 900 cases of *C difficile* infection (CDI) and 12 800 attributable deaths in 2017, the Centers for Disease Control and Prevention (CDC) considers *C difficile* an urgent threat [3]. An essential component to accurate reporting of CDI is an accurate diagnosis. While laboratory methods alone cannot diagnose CDI, they are integral for supporting a clinical diagnosis.

One of the main diagnostic tools used for *C difficile* is the polymerase chain reaction (PCR) assay for the presence or absence of the *tcdB* gene. The *tcdB* gene represents the presence or absence of the toxigenic locus, which is highly conserved among *C difficile* strains and encodes the toxin(s) responsible for symptomatic infection [4]. However, PCR testing does not differentiate between *C difficile* colonization and active infection. *Clostridioides difficile* is found in approximately 10% of patients [5], although some groups have colonization rates near 50% [6]. Colonized patients do not require treatment, and administration of vancomycin to colonized individuals typically does not resolve colonization nor decrease environmental contamination, although it may increase rates of vancomycin-resistant *Enterococcus* [7, 8].

PCR testing alone overdiagnoses CDI by an estimated 58% by failing to distinguish colonization from infection [9]. This in turn leads to overutilization of anti–*C difficile* therapy and delays in the diagnosis and treatment of the underlying cause of diarrhea. To overcome this challenge, current guidelines recommend multistep laboratory testing processes. The 2018 Infectious Diseases Society of America guidelines suggested a multiple testing approach, such as glutamate dehydrogenase (GDH) and a toxin test, arbitrated by a nucleic acid amplification test (such as PCR) for discordant results, or PCR followed by a toxin test [10]. The American College of Gastroenterology updated its guidelines for *C difficile* infections in 2021 to be in accordance with the European Society of Clinical Microbiology and Infectious Diseases, which recommends the use of a reverse 2-step algorithm where positive PCR testing is followed by stool toxin enzyme immunoassay (EIA) [11, 12].

**Figure 1.**
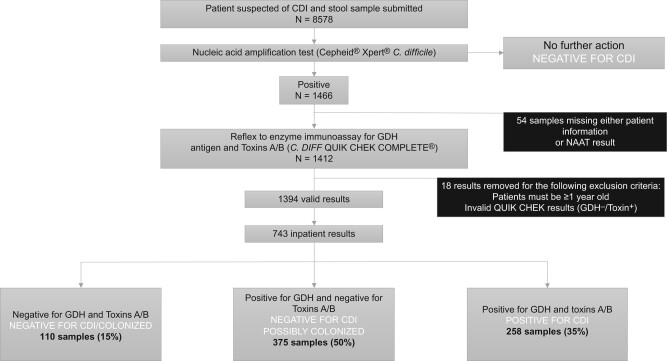
Outline of the reverse 2-step algorithm implemented at University of Minnesota Fairview. Patients with suspected *Clostridioides difficile* infection have a stool sample submitted to the clinical microbiology laboratory for testing. First the polymerase chain reaction test is run and if positive, reflex testing to the stool glutamate dehydrogenase (GDH) antigen and toxin enzyme immunoassay is automatically done. Numbers represent samples included in cycle threshold analysis. QUIK CHEK results with a negative GDH antigen and positive toxin were considered invalid and excluded. Abbreviations: CDI, *Clostridioides difficile* infection; GDH, glutamate dehydrogenase; NAAT, nucleic acid amplification test.

In October of 2022, the University of Minnesota Infectious Diseases Diagnostic Laboratory (IDDL) implemented a reverse 2-step algorithm for *C difficile* testing (Figure 1). All stool samples that were positive for *tcdB* by PCR were reflexed to the *C. DIFF* QUIK CHEK COMPLETE (Techlab, Blacksburg, Virginia), which reported on the presence or absence of the GDH enzyme and toxin A/B by EIA. The primary aim of this study was to describe the impact of the change in laboratory practice on hospital-onset (HO) CDI rates. We also explored the relationship between PCR cycle threshold (Ct) values and testing group based on GDH antigen and toxin EIA status.

## MATERIALS AND METHODS

We performed a quasi-experimental study of readily available de-identified data in our academic healthcare system to understand the impact of instituting reflex CDI testing. Two sources of data were available for use. First, we examined HO-CDI cases, as defined by the CDC's National Healthcare Safety Network (NHSN), which are tracked institutionally as part of routine quality measures. NHSN defines HO-CDI as a sample testing positive for CDI collected >3 days after admission. As part of this tracking, systemwide use of antibiotics for CDI (oral vancomycin and fidaxomicin) is also available. Second, we analyzed the Ct values of PCR^+^ samples from hospitalized patients after the institution of reflex CDI testing.

### Patient Population and Data Collection

For the HO-CDI rates, we selected a timeframe prior to include 7 months of data before the institution of reflex testing (1 March 2022 to 30 September 2023). Rates were obtained from the system in aggregate and reported as new cases per 1000 patient-days. Anti-infective therapeutic agents considered first-line for the treatment of acute CDI were tracked as part of routine monitoring by our health system's pharmacy department, and the days of therapy (DOT) per 1000 patient-days for enteral vancomycin and fidaxomicin were measured. To capture the number of instances providers intended to treat acute *C difficile* infection, we also measured the number of unique instances providers prescribed 4-times-daily oral vancomycin or twice-daily fidaxomicin. If an individual received these antibiotics on >1 occasion, they were included again if antibiotic initiation was >6 weeks from prior antibiotic use. Vancomycin use was solely at the discretion of the clinical team while fidaxomicin requires consultation with the antibiotic stewardship team.

For the Ct values, data were collected from inpatients who had stool testing that was PCR^+^ for *tcdB* between 18 October 2022 and 30 June 2023. The University of Minnesota IDDL processes stool samples for *Clostridioides difficile* testing from 8 hospitals in the M Health Fairview system, encompassing a mix of academic and community hospitals. Patients were included if >1 year of age and if the stool antigen and toxin EIA results were valid. Patient characteristics of age and location of collection were collected via the label that was submitted with the specimen to the clinical microbiology laboratory. Using the location of collection, we were able to identify samples sent from hospital-based locations, but as the label does not include the date of admission, we could not exclude samples sent within 3 days of admission. The results of the PCR and GDH enzyme and toxin A/B (toxin) by EIA were documented for each case.

### Reverse 2-Step Algorithm Tests

The PCR test used was the Cepheid Xpert *C difficile* PCR assay (Cepheid, Sunnyvale, California). In brief, this is a PCR assay that uses a test cartridge to directly test stool specimens for the toxin B gene (*tcdB*). The result in the patient's chart is a qualitative result of negative or positive for *C difficile tcdB* gene. Since it is a PCR assay, a Ct result is available to review in the laboratory.

Any positive in the Cepheid Xpert *C difficile* PCR assay was automatically reflexed to the stool antigen and toxin EIA, *C. DIFF* QUIK CHEK. In brief, a cassette is inoculated with stool, and an EIA detects both the GDH antigen and toxins A and B of *C difficile*. There are 4 possible test results of the *C. DIFF* QUIK CHEK that can lead to different interpretations (Figure 1). The first is GDH^–^/toxin^–^ and, as performed after a positive PCR, suggests colonization with toxigenic *C difficile*. The second is GDH^+^/toxin^−^, again suggesting the patient is colonized with toxigenic *C difficile*. The third result is GDH^+^/toxin^+^ and indicative of CDI. A testing pattern of GDH^−^/toxin^+^ is considered an invalid result on the testing manufacturer's instructions.

### Statistical Analysis

Categorical characteristics were summarized using counts and rates, and Ct values were summarized using median and interquartile range (IQR). Categorical characteristics were compared between groups using Fisher exact test. Ct values were compared between groups using the Wilcoxon rank-sum test. Receiver operating characteristic (ROC) analysis was used to examine the sensitivity and specificity for identifying GDH^+^/toxin^+^ and GDH^−^/toxin^−^ at various Ct thresholds. Youden index was used to identify the Ct threshold that maximized the sum of sensitivity and specificity. Analyses were conducted using R version 4.2.2 (R Foundation for Statistical Computing, Vienna, Austria).

### Patient Consent Statement

The design of the work has been approved by the University of Minnesota institutional review board (IRB). A waiver of patient consent was obtained by the IRB for the patient chart review.

## RESULTS

### HO-CDI Rates Before and After the Introduction of Reflex Testing

**Figure 2.**
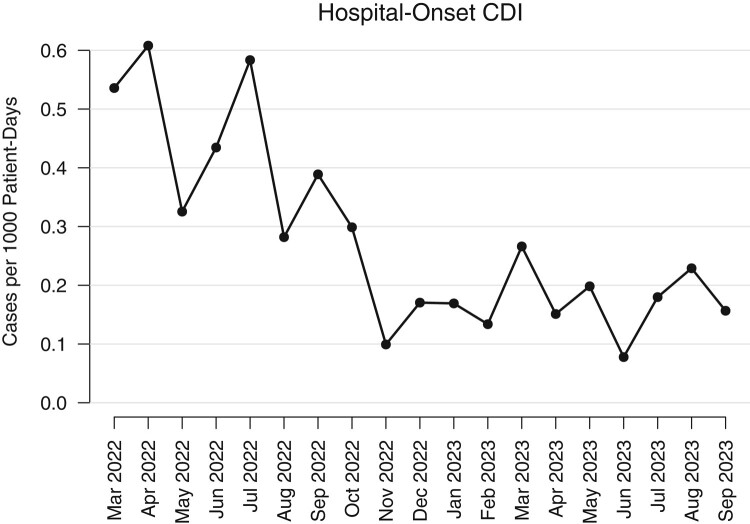
Cases of hospital-onset *Clostridioides difficile* infection (CDI) per 1000 patient-days from March 2022 through September 2023. The overall HO-CDI rate for the health system is displayed. Reverse 2-step reflex testing was initiated in October 2022.

Following the introduction of multistep testing, the systemwide HO-CDI rate decreased (Figure 2). The median 7-month rate of HO-CDI prior to the testing change (March 2022 through September 2022) was 0.352 cases per 1000 patient-days (IQR, 0.184–0.851). When examining the same period, the following year (March 2023 through September 2023), there was a significant decrease in the HO-CDI rate (median, 0.115 cases per 1000 patient-days [IQR, 0.064–0.133]; Wilcoxon *P* < .005). This corresponds to a 67% relative decrease in the rates of HO-CDI as defined by laboratory testing. HO-CDI rates decreased from March to September 2022 (prior to the October 2022 protocol change), but there was not sufficient evidence to conclude that this pattern was significant (rate of change, −0.03 cases per 1000 patient-days per month [95% confidence interval, −.09 to .03]; *P* = .25).

### Hospital Antibiotic Usage for CDI

**Figure 3.**
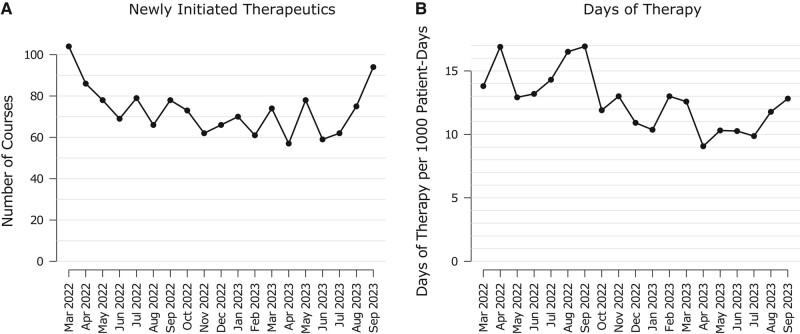
*A*, Instances of newly initiated oral vancomycin or fidaxomicin for acute *Clostridioides difficile* infection (March 2022 to September 2023), by month. *B*, Days of therapy of *C difficile* antibiotics (enteral vancomycin and fidaxomicin) per 1000 patient-days (March 2022 to September 2023), by month.

The instances of newly initiated enteral vancomycin (125 mg 4 times daily) or fidaxomicin and the DOT per 1000 patient-days for the same 2 agents from March 2022 through July 2023 are depicted in Figure 3. The frequency of new hospital-initiated orders of vancomycin and fidaxomicin was unchanged from March 2022 through September 2023. However, DOT decreased following the testing change. During the 7 months prior to the testing change (March 2022 through September 2022), the median DOT per 1000 patient-days was 14.3 (IQR, 13.5–16.7). When examining the same period, the following year (March 2023 through September 2023), the median DOT per 100 patient-days was 10.3 (IQR, 10.0–12.2), a significant decrease (Wilcoxon *P* = .0006). This corresponds to a 28% relative decrease in anti-CDI antibiotics.

### Ct Values of PCR-Positive Samples

Between 18 October 2022 and 30 June 2023, a total of 8578 unformed stools samples were submitted to the clinical microbiology laboratory for suspected CDI (Figure 1). Formed stool samples are routinely rejected by the laboratory. Seventeen percent (1466/8578) of PCR tests returned positive. Fifty-four samples were excluded due to missing patient information or a missing PCR result. Eight results were excluded due to patients being aged <1 year, and 10 were excluded for an invalid EIA result (GDH^−^/toxin^+^), resulting in 1394 samples in our system that underwent analysis of the reverse 2-step testing. Of these tests, 743 (53%) were performed in the hospital setting (including the emergency department). Of the 743 PCR^+^ hospital tests, 258 (35%) demonstrated concordance with both GDH and toxin positivity; 375 (50%) tests were GDH^+^ but toxin negative, and 110 (15%) were negative for both GDH and toxin. PCR^+^ hospitalized patients were less likely to have a positive toxin EIA relative to PCR^+^ outpatients (35% vs 40%, *P* = .04).

**Figure 4.**
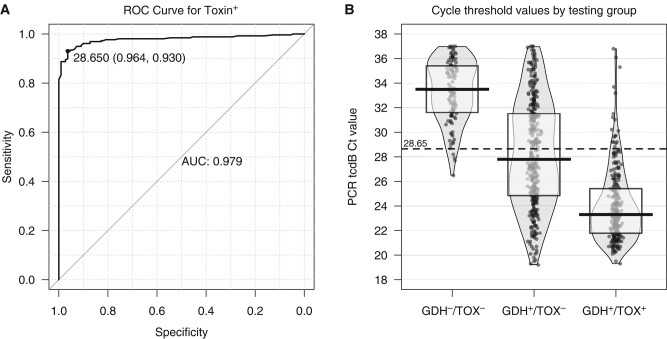
*A*, Receiver operating characteristic (ROC) analysis for toxin-positive enzyme immunoassay based on results that were glutamate dehydrogenase (GHD)**^−^**/toxin**^−^** and GDH^+^/toxin^+^. *B*, Distribution of *C. DIFF* QUIK CHEK results versus the Xpert *C difficile* polymerase chain reaction assay toxin cycle threshold (Ct) result with ROC threshold added (red dashed line). Ct values were significantly different for each group. Solid lines and boxes indicate the median and interquartile range in each group. Abbreviations: AUC, area under the curve; Ct, cycle threshold; GDH, glutamate dehydrogenase; PCR, polymerase chain reaction; ROC, receiver operating characteristic; TOX, toxin.

Among PCR^+^ specimens from hospital patients, the median Ct value of the GDH^−^/toxin^−^ group was 33.5 (IQR, 31.6–35.4), while the mean Ct value of the GDH^+^/toxin^+^ group was significantly lower at 23.3 (IQR, 21.8–25.4) (*P* < .0001). Specimens that were PCR^+^/GDH^–^/toxin^–^ likely reflect individuals colonized with *C difficile* while those that were PCR^+^/GDH^+^/toxin^+^ likely had CDI. We therefore used these 2 populations of hospital specimens to develop an ROC curve to identify a Ct threshold to distinguish colonization from infection. We found that a Ct threshold of 28.65 resulted in a sensitivity of 93.0% and a specificity of 96.4% with an area under the curve of 0.979 (Figure 4*A*). Cases where Ct failed to accurately predict toxin were further explored.

Four individuals were predicted to be toxin positive based on a Ct value of ≤28.65 but were GDH and toxin negative. All individuals had new-onset diarrhea and recent antibiotic use as well as an alternative plausible cause of diarrhea. Most were female (3/4), and most were aged <65 years (3/4). Two individuals were treated for CDI: 1 had repeat CDI testing 2 days later that was positive for toxin by EIA, prompting CDI treatment. Her posthospital course was complicated by recurrent, fulminant CDI requiring a colectomy. Another individual treated for CDI was not retested during an uneventful hospital course. Of the 2 individuals not treated for CDI, 1 presented to the hospital with severe sepsis attributed to a leg infection, was treated with piperacillin-tazobactam followed by ertapenem, and underwent *C difficile* testing 12 days later that yielded a PCR-negative result. The final individual had a single day of diarrhea that self-resolved.

Eighteen individuals were predicted not to have CDI based on a Ct value of >28.65 but were GDH^+^/toxin^+^; 17 of these individuals were treated for CDI. Sixty-seven percent (12/18) were over age 65 and 44% (8/18) were female. Three patients (17%) did not have new-onset diarrhea. One had a J-pouch with unchanged chronic diarrhea, while 2 others had CT findings of colitis without new-onset diarrhea. Nine patients (50%) met the criteria for severe CDI at the time of testing based on white blood cell count >15 000 cells/µL or creatinine >1.5 mg/dL (without underlying chronic kidney disease). Ninety-four percent (17/18) had recent antibiotic exposure and 62% (10/16) had another reasonable cause of diarrhea at the time of testing. The 1 individual who was not treated was asymptomatic and tested as part of a routine screening program on the bone marrow transplant unit. Nine individuals were retested for *C difficile* with 67% (6/9) testing PCR^+^/GDH^+^/toxin^+^. The initial *tcdB* PCR Ct values were numerically lower for those who retested with a PCR^+^/GDH^+^/toxin^+^ compared to those who retested PCR^+^/GDH^+^/toxin^−^ (n = 1) or were not retested (n = 11).

### Cycle Threshold Values of Discordant Testing Results

Among PCR^+^ specimens, discordant testing likely reflects a mixture of individuals colonized with *C difficile* and individuals with CDI. Consistent with this was a median Ct value of 27.8 (IQR, 24.8–31.5) for individuals testing GDH^+^/toxin^−^, a value in between those who were GDH^−^/toxin^−^ and those who were GDH^+^/toxin^+^. Applying the threshold identified from the ROC analysis, 54% of individuals testing GDH^+^/toxin^−^ had a Ct value ≤28.65, suggesting they might have CDI with a negative toxin EIA (Figure 4*B* and Table 1).

**Table 1.** Proportion of samples predicted to have Clostridioides difficile infection based on cycle threshold results across toxin group

Ct Value	GDH^–^/Toxin^–^ (n = 110)	GDH^+^/Toxin^–^ (n = 375)	GDH^+^/Toxin^+^ (n = 258)	*P* Value^a^
≤28.65	4 (4%)	204 (54%)	240 (93%)	<.0001

Abbreviations: Ct, cycle threshold; GDH, glutamate dehydrogenase.

^a^Pearson χ^2^ test across all groups.

## DISCUSSSION

Interventions to enhance testing stewardship, including optimizing laboratory diagnostics, can benefit the healthcare system by minimizing overdiagnosis and overtreatment. Following the introduction of reverse multistep laboratory reflex testing in the clinical microbiological laboratory in our healthcare system, reportable HO-CDI rates decreased by approximately 67%. This is consistent with prior literature suggesting that >50% of PCR^+^  *C difficile* tests do not have detectable toxin by EIA. HO-CDI rates are an important quality metric, publicly reported, and tied to quality-based reimbursements from Medicare. Antibiotic use is a product of providers interpreting laboratory data in the context of a patient's clinical situation. We observed a decrease in DOT for anti-CDI agents following the implementation of reflex testing. We hypothesize that providers reevaluated and stopped treatment for patients who were PCR^+^/toxin^−^ and had a low clinical likelihood of CDI. This finding is consistent with larger studies examining DOT and multistep CDI testing [13]. While CDI is not a laboratory diagnosis, rather a clinical one, the available data suggest that an institutional change to reflex laboratory testing will decrease anti-CDI antibiotic use. However, we found that the decrease in anti-CDI antibiotic use was not commensurate with the decrease in HO-CDI rates, suggesting that many providers elected to treat PCR^+^/toxin^−^ patients for CDI, illustrating the complexities of an accurate CDI diagnosis and the limitations of overreliance on laboratory diagnostic measures. In other studies, 75%–90% of PCR^+^/toxin^–^ patients receive at least partial treatment for CDI [14, 15].

Discordant results are the most common outcome of reflex *C difficile* testing [9, 16]. In our cohort, 65% had discordant PCR and toxin results with 15% having discordant PCR and GDH results and 50% having discordant GDH and toxin results. Individuals with discordant CDI testing are a particular challenge as they reflect a mixture of colonized and infected individuals. Incorporating Ct values into the interpretation of samples whose PCR/toxin results are discordant is a tempting endeavor, but ultimately Ct values are not ideal to use clinically due to the many variables that can contribute to the results. These include the collection method, the time from collection of the specimen to the testing, the type of instrument used, the methodology, and the Ct variation that can occur between and within methods [17]. From our limited review of data, 50% (2/4) of individuals who were toxin negative but predicted to have toxin based on Ct threshold likely did not have CDI based on available data in the chart. Additionally, toxin testing is fallible as asymptomatic carriers may have high stool toxin concentrations [18], and individuals with severe or fulminant CDI may test toxin negative. Providers should thus be aware that potentially half of discordant tests may represent infection. Appropriate testing and clinical acumen thus remain the cornerstone for an appropriate CDI diagnosis.

Our findings support the movement toward multistep laboratory CDI diagnostic testing to curb the overdiagnosis of publicly reported CDI and to decrease the treatment of likely colonized individuals. While multistep laboratory testing can be performed in a variety of ways [19], we advocate for implementing reflex EIA testing following PCR testing. In our cohort, 15% of *tcdB* PCR^+^ patients were GDH^–^. If screening relied only on GDH, these individuals would not have been identified. These individuals may be colonized with *C difficile* and evidence suggests that hospital patients with diarrhea who are colonized with toxigenic *C difficile* are a reservoir for transmission and require infection control precautions [20]. In general, PCR testing has a superior sensitivity than GDH antigen testing and is therefore a potentially better initial test [21]. Some authorities recommend a combination GDH/toxin EIA as the initial test, and arbitrating discordant EIA results with PCR testing by regarding a positive subsequent PCR test as CDI. However, with this strategy no additional information on toxin production is obtained. Therefore, using PCR testing to arbitrate discordant EIA testing will perpetuate overdiagnosis of CDI.

Implementing this strategy is feasible as most clinical laboratories have existing clinical infrastructure for PCR testing for *tcdB* or a combination of *tcdA* and *tcdB*. Reflexing to the *C. DIFF* QUIK CHEK, which is a stand-alone point of care lateral flow assay, does not require additional machinery to be added to the laboratory space. However, as the *C. DIFF* QUIK CHEK is a lateral flow cassette, there is some interpretation in positive and negative results. Therefore, technician training and protocols for ambiguous results are required. In our laboratory, 2 technologists read each *C. DIFF* QUIK CHEK device and initialed a label with the results of the 2 targets listed. Ambiguous results were mediated by senior technical staff and/or IDDL medical directors.

Our analysis has limitations. Our measurement of CDI rates relies upon aggregate system data and does not include non–HO-CDI. Our Ct value analysis is from an available dataset that is not linked to admission date. Thus, while the patient was at an inpatient hospital location, the Ct values reflect a more diverse group of patients, including samples sent as part of the diagnostic evaluation for people admitted for diarrhea. Our measurement of anti-CDI antibiotics initiation and DOT was not based on testing results, and therefore includes antibiotics started empirically (which may have later been stopped based on subsequent testing) and antibiotics continued from the outpatient setting. While we were able to perform a limited review of select charts to describe the clinical course of patients whose Ct values did not correctly predict the toxin status, a comprehensive review of the ≥760 inpatients was out of scope for this project. Undoubtably, patient characteristics at the time of testing and outcomes following testing are extremely important in understanding discordant tests. Without patient data, we were also unable to stratify by important subpopulations such as those with neutropenia, transplant populations, and inflammatory bowel disease—all cases where *C difficile* colonization and infection are diagnostic challenges. While we were able to collect Ct values, there are other CDI-related diagnostics that would be useful. A gold standard CDI test, such as toxigenic culture, would help in determining an accurate diagnosis in discordant testing. Additionally, ribotyping would be of interest, but is not routinely done in our laboratory. Future projects and data collection are underway to overcome these limitations.

In conclusion, our findings support the use of a reverse, 2-step testing algorithm for a laboratory diagnosis of CDI—specifically, a reflex GDH/toxin EIA for PCR-positive tests. We found a dramatic decrease in the rates of publicly reported HO-CDI postimplementation of the new laboratory testing algorithm along with a decrease in anti-CDI antibiotics as measured by DOT, although not CDI therapy initiation. The implementation of an EIA to a clinical microbiology laboratory using an existing PCR platform is feasible and has minimal impact on the laboratory space and workflow. With reflex testing, discordant PCR and toxin results are the most common. Ct values are unlikely to be clinically useful, but in aggregate suggest that those with discordant results are an even mix of colonized and infected individuals. Clinicians should be aware of the limitations of laboratory testing. Additional research is needed to distinguish infection from colonization in people with discordant CDI testing.

## Notes

***Acknowledgments.*** The authors would like to acknowledge Kimberly Boeser and Tami Dahlquist for sharing system-level pharmacy and infection control data. Also, we thank the technologists in the IDDL for performing these assays.

***Author contributions.*** E. E. H. and P. F. both contributed to the immunoassay implementation, study concept and design, data acquisition, interpretation of results, and writing and critical review of the manuscript. M. D. E. contributed to the statistical analysis, interpretation of results, and critical review of the manuscript. A. L. G. and B. P. V. contributed to the interpretation of the results and writing and critical review of the manuscript.

***Financial support*.** This research was supported by the National Institutes of Health's National Center for Advancing Translational Sciences (grant number UL1TR002494 to M. D. E.).
